# Supplementary material for: Unveiling hidden energy poverty using the energy equity gap
Source: Nat Commun. 2022 May 4;13:2456. doi: 10.1038/s41467-022-30146-5 (PMC9068781; doi:10.1038/s41467-022-30146-5)
Supplement: Supplementary file 1 — Supplementary Information PDF [file 41467_2022_30146_MOESM1_ESM.pdf]

Supplementary Information

Unveiling Hidden Energy Poverty Using the Energy Equity Gap

Shuchen Cong[1], Destenie Nock[1, 2, *], Yueming (Lucy) Qiu[3, *], Bo Xing[4]

[1]Department of Engineering and Public Policy, Carnegie Mellon University, PA, USA

[2]Department of Civil and Environmental Engineering, Carnegie Mellon University, PA, USA

[3]School of Public Policy, University of Maryland College Park, College Park, MD, USA

[4]Department of Forecasting, Resource Planning and Development, Salt River Project, Tempe, AZ, USA

[*]Corresponding authors: Nock dnock@andrew.cmu.edu ; Qiu yqiu16@umd.edu

Supplementary Figures

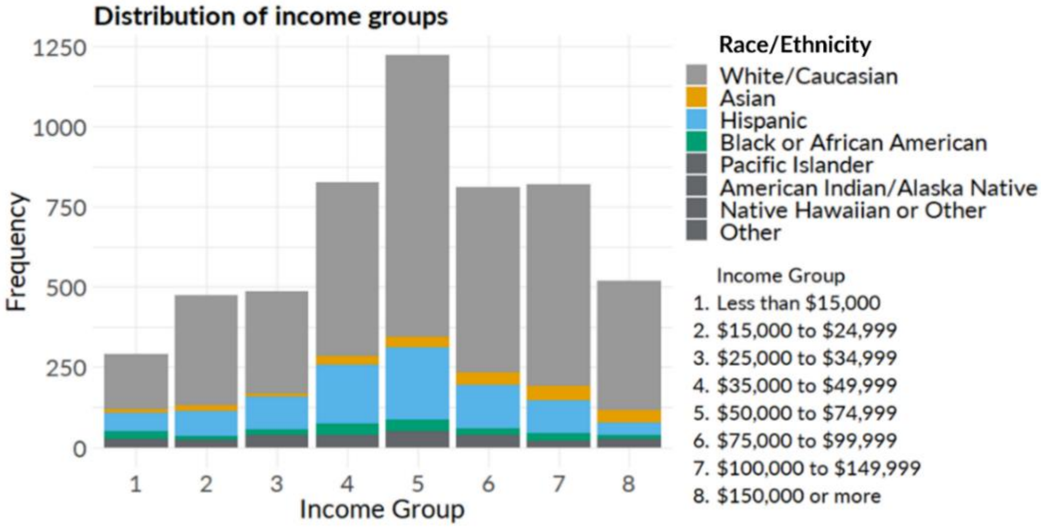

Supplementary Figure 1. Racial and ethnic distribution by income group within our sample from Arizona.

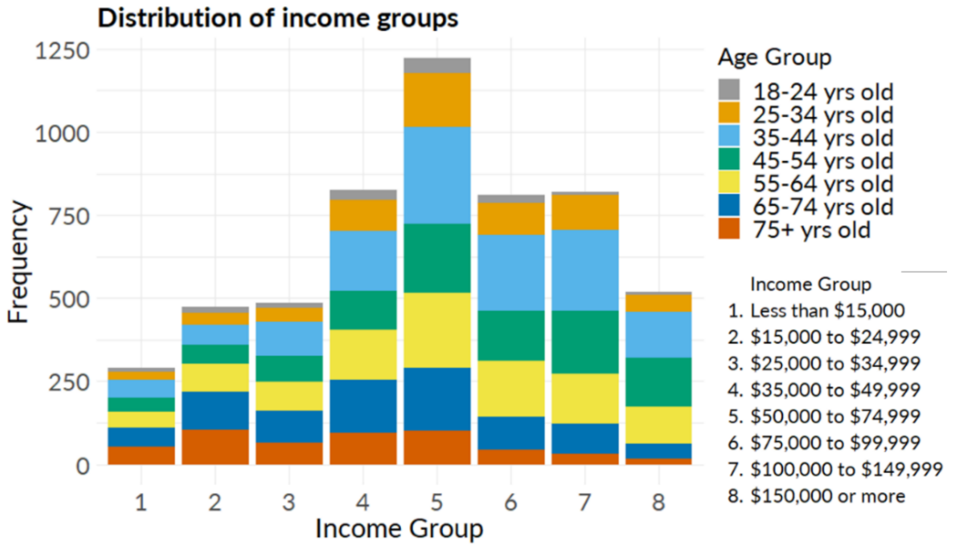

Supplementary Figure 2. Age distribution by income group within the dataset.

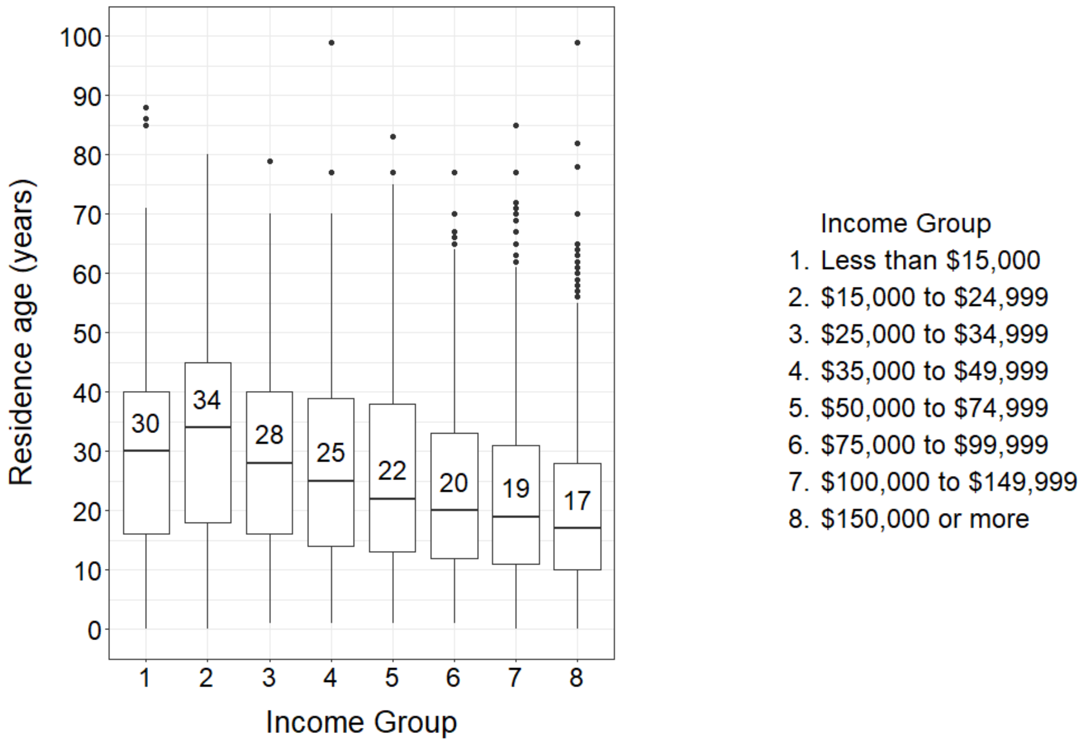

Supplementary Figure 3. Residence age distribution by income group within the dataset. N = 4,700. We note that lower income groups tend to have older residences. Each box and whiskers plot indicates the minima and maxima of residence ages of one income group (the lower and upper bound of the whiskers), the first and third quantiles (the lower and upper bound of the box), and the median (the middle line). The outliers are shown as dots on either side of the whiskers. Source data can be found in our code repository.

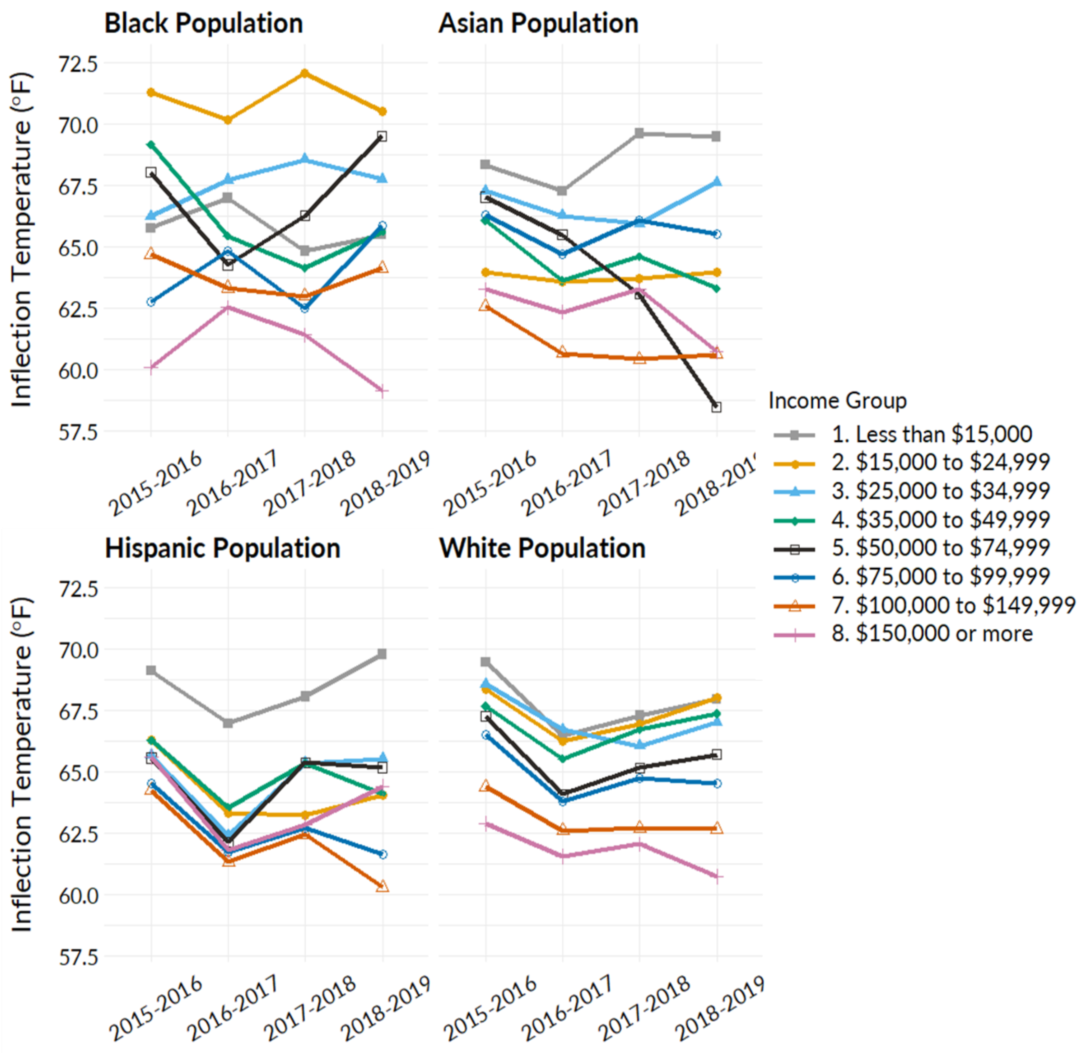

Supplementary Figure 4. Energy equity gap within ethnicities

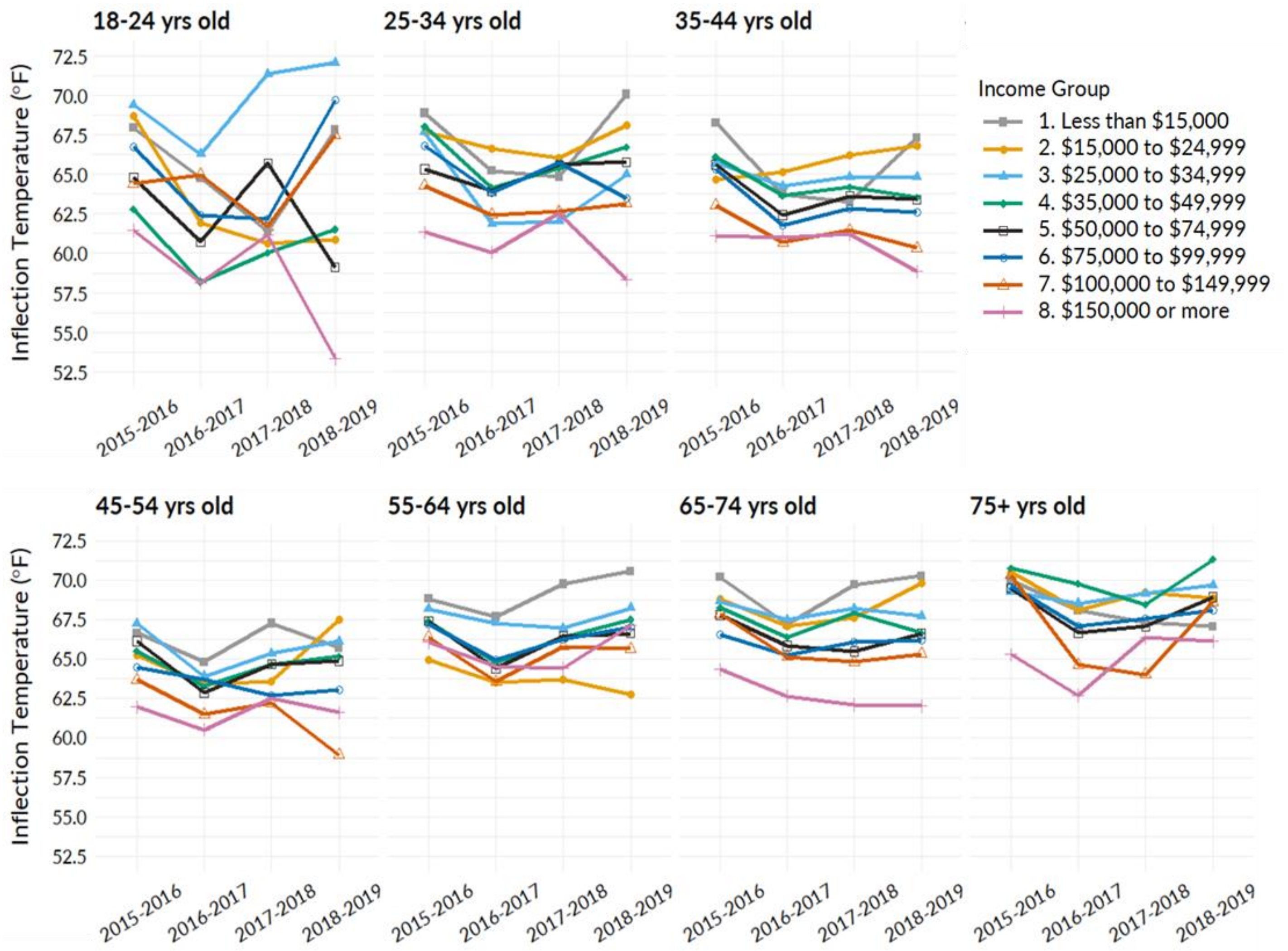

Supplementary Figure 5. Energy equity gap within age groups

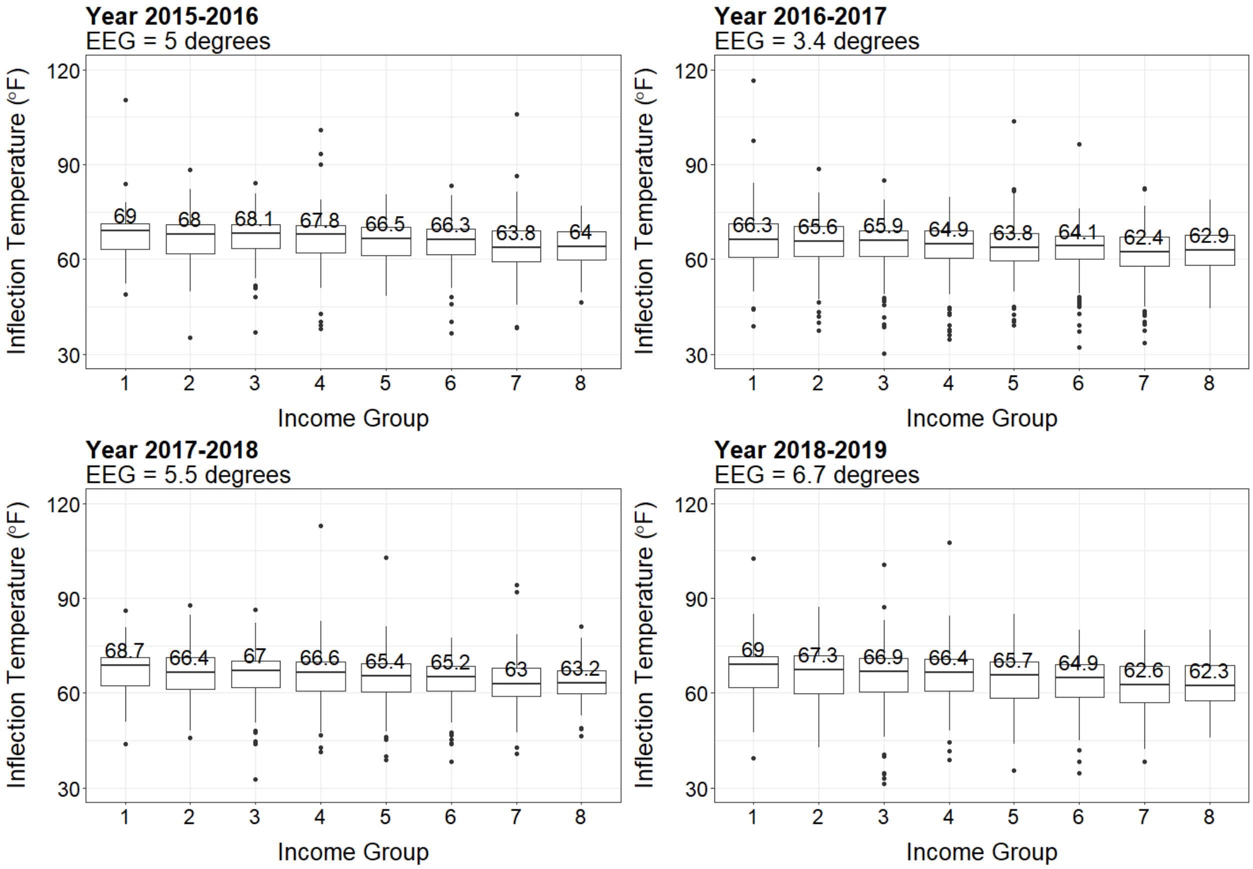

Supplementary Figure 6. Inflection temperature calculated for households present in all four years of data, n = 1,984 for all years. Each box and whiskers plot indicates the minima and maxima of inflection temperatures of one income group for one year (the lower and upper bound of the whiskers), the first and third quantiles (the lower and upper bound of the box), and the median (the middle line). The outliers are shown as dots on either side of the whiskers. Source data can be found in our code repository.

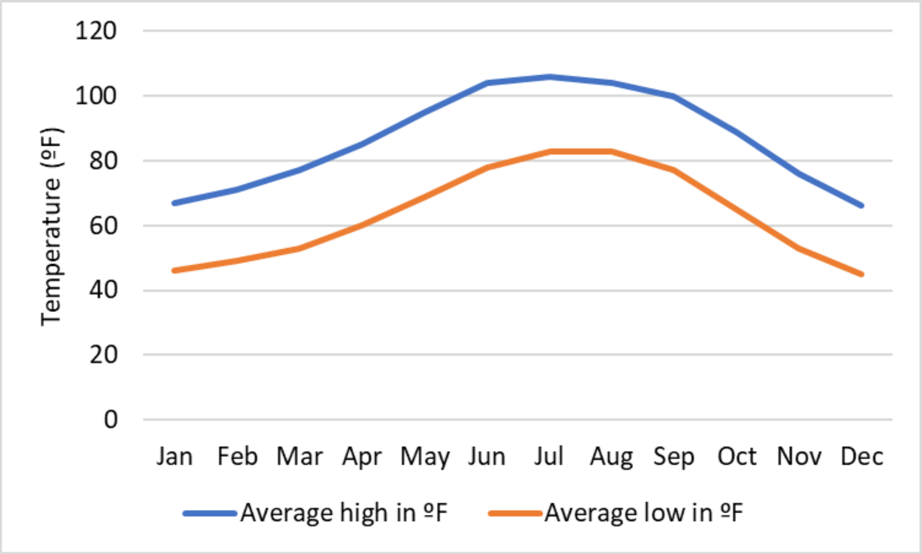

Supplementary Figure 7. Monthly average temperatures in the study area, data taken from usclimatedata.com[2]

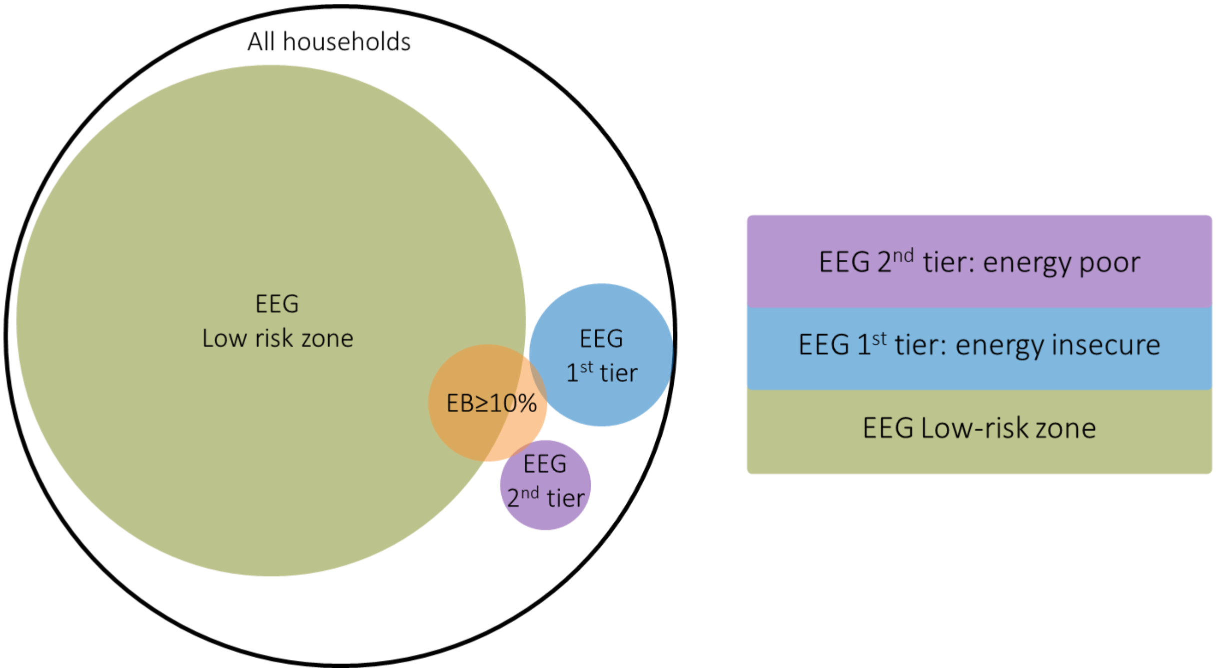

Supplementary Figure 8. Venn diagram representation of the number of households captured by the energy equity gap (EEG) vs. energy burden (EB), 2015-2016. EEG 1st tier is energy insecurity, EEG 2nd tier is energy poverty. Only three households overlap between EEG 2nd tier (energy poor) and those with an energy burden greater than 10%.

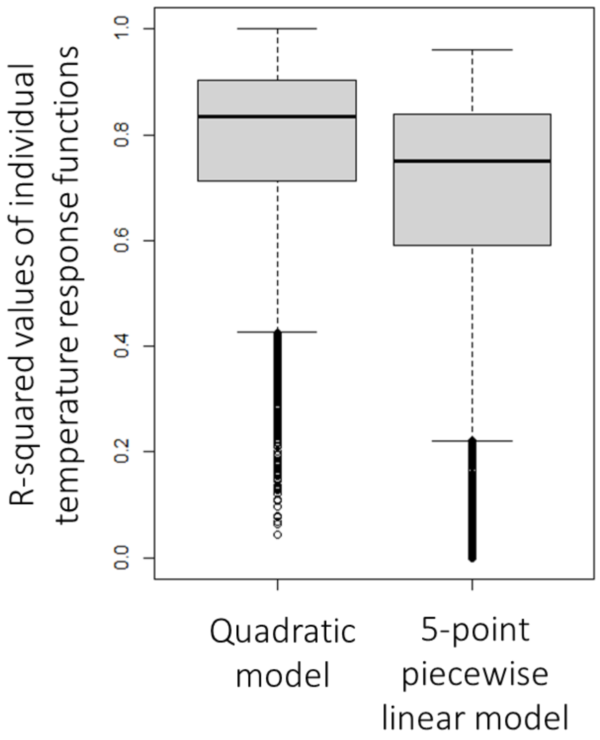

Supplementary Figure 9. A comparison of R2 values of household temperature response functions using two models: the quadratic temperature response function used to calculate inflection temperatures in this paper (see Equation 1 in the main text), and a 5-point piecewise linear function that calculates a separate heating and cooling inflection points. N = 4104 for both models. Each box and whiskers plot indicates the minima and maxima of r—squared values of each model (the lower and upper bound of the whiskers), the first and third quantiles (the lower and upper bound of the box), and the median (the middle line). The outliers are shown as dots on either side of the whiskers. Source data can be found in our code repository.

Supplementary Tables

Supplementary Table 1. Linear regression output of different variables against household inflection temperature in 2015-2016. The intercept represents multi-family residence type, zero years of residence age and zero people in the household, residences less than 1000 sqft, income less than $15,000, and with one AC unit. VIF is the variance inflation factor. CONDO, MOBILE_HOME, SINGLE_FAMILY_HOME, and TOWNHOUSE are the other types of residences; RESIDENCE_AGE_INTEGER is the age of the building in integer years; HOUSEHOLD_SIZE_INTEGER is the number of people in the household; SIZE_SQFEET variables are the brackets for the size of residence in square feet; INCOME variables are income brackets; ACUNITS are the number of AC units a household has. Two-sided t-tests are conducted to test whether the coefficients are statistically significantly different from zero. No adjustments were made for multiple comparisons.

Coefficients:	Estimate	Std. Error	t value	Pr(>|t|)		VIF
(Intercept)	66.36236	0.739378	89.754	< 2e-16	***	
CONDO	1.694445	0.712611	2.378	0.01747	*	1.87
MOBILE_HOME	1.474602	0.721836	2.043	0.04114	*	1.86
SINGLE_FAMILY_HOME	-0.78091	0.535774	-1.458	0.14506		4.06
TOWNHOUSE	0.732204	0.674062	1.086	0.27744		2.20
RESIDENCE_AGE_INTEGER	0.085387	0.006404	13.333	< 2e-16	***	1.11
HOUSEHOLD_SIZE_INTEGER	-0.56163	0.070076	-8.015	1.48E-15	***	1.13
SIZE_SQFEET_1000_1499	-0.20266	0.438831	-0.462	0.64424		3.79
SIZE_SQFEET_1500_1999	0.251433	0.447885	0.561	0.57457		4.74
SIZE_SQFEET_2000_2999	-0.01388	0.495039	-0.028	0.97763		4.98
SIZE_SQFEET_3000_3999	0.133145	0.637963	0.209	0.83469		2.95
SIZE_SQFEET_4000_PLUS	0.419606	0.87177	0.481	0.63031		2.29
INCOME_15000_24999	-0.64764	0.618048	-1.048	0.29476		2.76
INCOME_25000_34999	-0.18295	0.609811	-0.3	0.76418		2.97
INCOME_35000_49999	-0.53293	0.572424	-0.931	0.35192		4.31
INCOME_50000_74999	-0.946	0.55598	-1.701	0.08894	.	5.71
INCOME_75000_99999	-1.16608	0.575186	-2.027	0.0427	*	4.74
INCOME_100000_149999	-1.8975	0.577287	-3.287	0.00102	**	5.07

INCOME_150000_PLUS	-2.504	0.614387	-4.076	4.69E-05	***	4.10
ACUNITS_TWO	0.021326	0.315746	0.068	0.94615		1.88
ACUNITS_THREE_PLUS	0.662092	0.721987	0.917	0.35918		1.67

Signif. codes: 0 ‘***’ 0.001 ‘**’ 0.01 ‘*’ 0.05 ‘.’ 0.1 ‘ ’ 1

Supplementary Table 2. Linear regression output of different variables against household income group. The intercept represents multi-family residence type, zero years of residence age and zero people in the household, residences less than 1000 sqft, and with one AC unit. CONDO, MOBILE_HOME, SINGLE_FAMILY_HOME, and TOWNHOUSE are the other types of residences; RESIDENCE_AGE_INTEGER is the age of the building in integer years; HOUSEHOLD_SIZE_INTEGER is the number of people in the household; SIZE_SQFEET variables are the brackets for the size of residence in square feet; ACUNITS are the number of AC units a household has; ETHNIC variables are ethnicities, AGE_INT is the integer age of the head of household. Two-sided t-tests are conducted to test whether the coefficients are statistically significantly different from zero. No adjustments were made for multiple comparisons.

Coefficients:	Estimate	Std. Error	t value	Pr(>|t|)	
(Intercept)	5.431692	0.198677	27.339	<2e-16	***
CONDO	-0.01358	0.194364	-0.07	0.944299	
MOBILE_HOME	-0.71196	0.198194	-3.592	0.000332	***
SINGLE_FAMILY_HOME	0.262174	0.146813	1.786	0.074222	.
TOWNHOUSE	0.274148	0.184649	1.485	0.137711	
RESIDENCE_AGE_INTEGER	-0.00414	0.001766	-2.346	0.019037	*
HOUSEHOLD_SIZE_INTEGER	-0.01057	0.021225	-0.498	0.618596	
SIZE_SQFEET_1000_1499	0.771301	0.118426	6.513	8.40E-11	***
SIZE_SQFEET_1500_1999	1.342806	0.119969	11.193	<2e-16	***
SIZE_SQFEET_2000_2999	1.93462	0.131847	14.673	<2e-16	***
SIZE_SQFEET_3000_3999	2.204995	0.170755	12.913	<2e-16	***
SIZE_SQFEET_4000_PLUS	2.568061	0.233134	11.015	<2e-16	***
ACUNITS_TWO	0.482717	0.085949	5.616	2.10E-08	***
ACUNITS_THREE_PLUS	0.768275	0.197409	3.892	0.000101	***
ETHNIC_ASIAN	-0.1631	0.142514	-1.144	0.252515	
ETHNIC_BLACKA	-0.32426	0.186556	-1.738	0.08227	.

ETHNIC_HISP	-0.55496	0.07692	-7.215	6.57E-13	***
ETHNIC_OTHER	-0.4591	0.168757	-2.72	0.00655	**
AGE_INT	-0.03106	0.001986	-15.64	<2e-16	***

Signif. codes: 0 ‘***’ 0.001 ‘**’ 0.01 ‘*’ 0.05 ‘.’ 0.1 ‘ ’ 1

Supplementary Table 3. Coefficients of regression model for calculating inflection temperatures. The following factors were added to Equation 1 in the main text and did not change nor improve model fit: home heating type (VHOMEHEAT), home AC type (VACTYPE), number of AC units (VACUNITS), residence size (VSQFEET), residence age(VRESAGE), age of the head of household(VHHAGE), the number of people in the household (VHOUSEHOLD_INT), and household income (VINCOME). All but residence size (VSQFEET), residence age(VRESAGE), age of the head of household(VHHAGE), the number of people in the household(VHOUSEHOLD_INT), and household income (VINCOME) are dummy variables. R squared and the coefficients of the original factors included in the model have not changed (coefficients of holidays, weekly and monthly fixed effects are not shown)

Household	r_squared	Coefficients														
		B_temp_avg	B_temp_avg_sq	B_elec_cost	B_VHOMEHEAT_ELEC	B_VACTYPE_GAS_HEAT	B_VACUNITS_TWO	B_VACUNITS_THREE_PLUS	B_VSQFEET_1000_1499	B_VSQFEET_1500_1999	B_VSQFEET_2000_2999	B_VSQFEET_3000_3999	B_VHOUSEHOLD_INT	B_VRESAGE	B_VHHAGE	B_VINCOME
Test 1	0.85	-3.85	0.03	-242.88	0	0	0	0	0	0	0	0	0	0	0	0
Test 2	0.86	-3.57	0.03	0.23	0	0	0	0	0	0	0	0	0	0	0	0
Test 3	0.81	-0.35	0.00	24.46	0	0	0	0	0	0	0	0	0	0	0	0
Test 4	0.83	-4.57	0.03	-265.03	0	0	0	0	0	0	0	0	0	0	0	0
Test 5	0.86	-11.41	0.08	-67.61	0	0	0	0	0	0	0	0	0	0	0	0
Test 6	0.82	-4.18	0.03	-3.24	0	0	0	0	0	0	0	0	0	0	0	0
Test 7	0.81	-3.33	0.03	1.67	0	0	0	0	0	0	0	0	0	0	0	0
Test 8	0.83	-6.40	0.04	-0.74	0	0	0	0	0	0	0	0	0	0	0	0

Supplementary Table 4. P-value matrix of two-tailed pairwise median test of inflection temperatures across income groups in 2015-2016. Alpha = 0.05.

	Less than $15,000	$15,000 to $24,999	$25,000 to $34,999	$35,000 to $49,999	$50,000 to $74,999	$75,000 to $99,999	$100,000 to $149,999
$15,000 to $24,999	5.71E-02						
$25,000 to $34,999	5.71E-02	9.45E-01					
$35,000 to $49,999	1.53E-02	2.98E-01	2.55E-01				
$50,000 to $74,999	1.70E-04	6.19E-02	2.82E-02	1.55E-01			
$75,000 to $99,999	6.20E-06	1.17E-02	4.02E-03	2.01E-02	2.21E-01		
$100,000 to $149,999	5.89E-10	3.69E-06	5.43E-08	7.14E-09	1.36E-06	4.05E-04	
$150,000 or more	2.43E-12	2.27E-09	2.43E-12	3.91E-12	4.88E-10	2.20E-07	1.53E-02

Supplementary Table 5. P-value matrix of two-tailed pairwise median test of inflection temperatures across age groups in 2015-2016. Alpha = 0.05.

	18-24	25-34	35-44	45-54	55-64	65-74
25-34	7.96E-01					
35-44	6.69E-01	1.67E-02				
45-54	6.69E-01	3.37E-02	7.97E-01			
55-64	3.59E-02	1.25E-02	3.29E-09	3.36E-09		
65-74	8.13E-03	2.65E-04	6.75E-11	5.34E-11	1.74E-02	
75+	2.19E-05	3.16E-13	1.18E-20	7.19E-21	3.48E-10	3.18E-05

Supplementary Table 6. P-value matrix of two-tailed pairwise median test of inflection temperatures across ethnicity groups in 2015-2016. Alpha = 0.05.

	White	Asian	Hispanic
Asian	0.27		
Hispanic	0.04945	0.4585	
Black	1	0.27	0.5808

Supplemental Information Note 1: Income, ethnicity, and age do not correlate in our dataset

Supplementary Figures 1 and 2 provide an overview of the demographic composition in our dataset. Along with Supplementary Tables 4 and 5, we show that income groups, age groups, and ethnicities are although correlated, their effect on each other is small.

Supplemental Information Note 2: Lower income groups have older homes

The variability in residence age across income groups is displayed in Supplementary Figure 3. Here we find that lower income groups tend to have older residences, with the median house age greater than thirty years old. While the higher income group tends to reside in newer homes, with the highest income groups residing in homes with a median age of less than 20 years.

Supplemental Information Note 3: Black population experiences the most inequity in energy usage

Supplementary Figure 4 shows the breakdown of median inflection temperatures by ethnicities by year. We see large inequalities within the black population from the wide vertical spread of the median inflection temperatures. The lowest income group in the black population may be receiving aid, lowering their inflection temperature below income group 2. We also see the lowest income group in the Hispanic population is even more worse off compared to the other ethnicities. We do not see a discernable difference in temperature preference across ethnicities, so ethnicity is not statistically a good indicator of inflection temperature. We want to note that the limited data for Black and Asian populations may contribute to uncertainty.

Supplemental Information Note 4: Age could indicate temperature preference, need to distinguish between preference for warm temperatures and energy limiting behavior

Supplementary Figure 5 compares the energy equity gap across age groups. There are statistically significant differences between median inflection temperatures across age groups (see table 3 in the main text), indicating that there is little chance that these variations occur solely due to chance. For the youngest group, the energy equity gap increased sharply (>14°F) between years 3 and 4, while for the older populations, the energy equity gap had little change in comparison. From an energy poverty targeting standpoint, this highlights that within the elderly population, all residents should be

targeted to reduce inflection temperatures, while for the youngest age groups, the most effective poverty eradication policy would be to target low-income groups.

For all age groups except for the oldest, the later increase in the energy equity gap is from low-income households getting worse off and high-income households performing better, most evident in groups 18-24 and 25-34. The difference in inflection temperatures between age groups may be attributed to each age group's different temperature comfort levels. Elders may prefer warmer indoor temperatures, and cooling air from an AC system may inflame arthritis, but we should be very cautious when differentiating between a comfortably warm temperature and one that puts the resident at risk for a heat-related illness[1].

Supplemental Information Note 5: Energy equity gap analysis for a subset of data

We present an analysis of the energy equity gap across a subset of households in Supplementary Figure 6. This subset represents the set of households who were present for all four years of our study. We find similar trends to the analysis for the full sample. The energy equity gap in the first year of the study is 5.9°F (3.3°C), and then in the second year of the study this drops to 4.7°F (2.6°C), but we see the EEG rises to 7.5°F (4.2°C) in the four years of our analysis. We see that these large shifts in the energy equity gap are directly related to the fluctuations in the lowest income group.

Supplemental Information Note 6: Arizona temperatures

Supplementary Figure 7 shows the monthly average high and low temperatures in Arizona, one of the warmest states in the US. The temperature usually peaks around July and August.

Supplemental Information Note 7: How many households can the energy equity gap capture in addition to those with high energy burden?

Supplementary Figure 8 shows a proportionate Venn diagram of households in each of the energy equity gap zone (low risk, energy insecure, and energy poor), and households with an energy burden over 10%. The corresponding data can be found in Table 2 of the main text.

Supplemental Information Note 8: Regression between inflection temperatures, housing characteristics and demographics

To understand the relationship between household level inflection temperatures and housing characteristics, as well as the relationship between income and housing characteristics and demographics, in our secondary regression, we first regressed household inflection temperatures against income groups and other variables (type of residence [i.e., single-family home, multi-family home, condo, mobile home, townhouse], residence age, residence size, number of AC units, ethnicity, and age). We find that income is correlated with all five variables (Supplementary Table 2). Thus, because these variables are not independent of each other, including income, the type of residence, residence age, residence size, number of AC units, ethnicity, and age in one analysis will introduce multicollinearity into the model, which interfere could influence our estimate of the relationship between income and inflection temperatures.

To further confirm this finding, we use the variance inflation factor (VIF). Supplementary Table 1 displays the regression output with inflection temperature as the dependent variable, and type of residence, residence age, residence size as the independent variables, along with variance inflation factor for each independent variable. The variance inflation factor provides an indication of whether an independent variable is correlated with another variable in the same regression model. When the variance inflation factor has a value of 1, the variable is not correlated with any other variable. The higher the variance inflation factor, the higher the chance of introducing multicollinearity into the model when both variables are included. We see that the residence size and income dummy variables have variance inflation factors close to or larger than 5, meaning including them in the same model would be double counting their effects on the dependent variable, the inflection temperature. From these findings, we conclude that using income as the only dependent variable to be compared to the inflection temperature is appropriate. Nonetheless, despite the collinearity issue, Supplementary Table 1 shows that the highest income groups have the lowest inflection temperatures (as reflected by the negative coefficients), which is consistent with our main conclusion.

Supplemental Information Note 9: Preferences versus inequities

When it comes to comfortable temperature preferences across different groups of people and cultures, the lack of thermostat data for the households in this study makes preference difficult to

gauge. In addition, the data only records the demographics of one person filling out the survey, meaning it does not capture multiracial families. Our objective is to provide utility companies with a method to identify energy-limiting behavior in different demographic groups. The electricity utility company we collaborated with collected electricity consumption data at the household level, but does not have information on electricity consumption by appliance. We acknowledge that there will be varying electricity consumption preferences across different demographics, but we note that preferences are influenced by a number of factors (e.g., perceived amount of spending available, body mass index, preferred climate, etc.). In our distinctions we see a wide variation within income groups (Figures 5b and 5d) as well as most racial, ethnicity and age groups. However, when investigating the groups as a whole, we find low-income groups are as a whole waiting longer to start using AC systems in the summer (i.e., higher inflection temperatures. We have investigated preferences of AC consumption across ethnicity and age groups. For example, Figures 5a and 5c illustrate the potential difference in inflection temperature preference between ethnicity and age groups, where Black and Asian groups may be less tolerant of heat, therefore have lower inflection temperatures overall. Similar with age groups, younger groups may prefer colder temperatures and older groups may prefer slightly warmer temperatures. Figures 5b and 5d illustrate the inequality within each ethnicity and age group, where Black and young households display the highest amount of inequality.

Supplementary Information Note 10: Including housing characteristics in the temperature response function

To confirm that housing characteristics do not affect household-level inflection temperature, we fitted a separate temperature response function to include housing characteristics, specifically residence age, type of residence, and residence size into the temperature response function, and found that they did not influence household-level inflection temperatures for the reason that they are household specific, and uniform across all data points for each particular household. Shown in Supplementary Table 3.

Supplemental Information Note 11: Pairwise median tests of median inflection temperatures grouped by income, age, and ethnicity

We also conducted post-hoc pairwise median tests to see how the median inflection temperature of each income/age/ethnicity group compares with each other (Supplementary Tables 4, 5,

and 6). For income groups, although some neighboring groups are not statistically different from each other, two to three groups over indicate strong evidence that the income groups have statistically different sample medians, where lower income groups have generally higher inflection temperatures, and higher income groups have lower inflection temperatures. Neighboring age groups are generally statistically significant from each other, besides the 18-24 and 35-44 groups. However, the general trend of younger groups having lower inflection temperatures and older groups having higher inflection temperatures still applies. There is the most uncertainty in the difference between ethnicities, which could be due to the limited data points for minorities, reflective of the study region.

Supplemental Information Note 12: Non-linear vs piecewise linear temperature response functions

Here we discuss the justification for the regression model used in our temperature response function analysis. Although piecewise linear temperature response functions are commonly used in the building literature[11–13], we chose to use a non-linear (i.e., quadratic equation) to model the electricity consumption response function to temperature, due to the latter giving a better fit (higher R^2 values, shown in Supplementary Figure 9) after testing both models. The piecewise linear model adapted from previous studies [14–16] gave an overall lower fit when compared to the quadratic equation. Here the median R^2 of the piecewise model lies around 0.75, while the median of the quadratic model is over 0.8. Additionally, within the quadratic 75% of households had a fit over 0.7. This finding of the nonlinear function producing a stronger fit is consistent with results from previous studies[13,17].

Supplementary References

1. Heat and Health. https://www.who.int/news-room/fact-sheets/detail/climate-change-heat-and-health.

2. Climate Phoenix - Arizona and Weather averages Phoenix. https://www.usclimatedata.com/climate/phoenix/arizona/united-states/usaz0166.

3. Lawes, M., Havenith, G. & Hodder, S. Ethnic differences: The influence of relative humidity on thermal perception. in *Comfort Congress 2021 Proceedings* (2021).

4. Wang, L., Chen, M. & Yang, J. Interindividual differences of male college students in thermal preference in winter. *Building and Environment* **173**, 106744 (2020).

5. Frederick H. Rohles, JR. Preference for the Thermal Environment by the Elderly: *https://doi.org/10.1177/001872086901100106* **11**, 37–41 (2016).

6. Li, P., Liu, Y. & Dong, J. Age-Related Thermal Comfort in a Science Museum with Hot–Humid Climate in Summer. *Environmental Science and Engineering* 421–431 (2019) doi:10.1007/978-981-13-9520-8_45.

7. Hwang, R.-L. & Chen, C.-P. Field study on behaviors and adaptation of elderly people and their thermal comfort requirements in residential environments. *Indoor Air* **20**, 235–245 (2010).

8. NA, T., NK, A. & DG, P. Preferred room temperature of young vs aged males: the influence of thermal sensation, thermal comfort, and affect. *The journals of gerontology. Series A, Biological sciences and medical sciences* **50**, (1995).

9. M, N. *et al.* Regional differences in temperature sensation and thermal comfort in humans. *Journal of applied physiology (Bethesda, Md. : 1985)* **105**, 1897–1906 (2008).

10. Natsume, K., Ogawa, T., Sugenoya, J., Ohnishi, N. & Imai, K. Preferred ambient temperature for old and young men in summer and winter. *International Journal of Biometeorology 1992 36:1* **36**, 1–4 (1992).

11. Woods, J. & Fuller, C. Estimating base temperatures in econometric models that include degree days. *Energy Economics* **45**, 166–171 (2014).

12. Perez, K. X., Cetin, K., Baldea, M. & Edgar, T. F. Development and analysis of residential change-point models from smart meter data. *Energy & Buildings* **139**, 351–359 (2017).

13. Fazeli, R., Ruth, M. & Davidsdottir, B. Temperature response functions for residential energy demand - A review of models. *Urban Climate* vol. 15 45–59 (2016).

14. Plagge, T., Ngo, P. & Gee, M. SAVINGS ESTIMATION USING THE OpenEEmeter. (2017).

15. EEmeter: tools for calculating metered energy savings — eemeter 3.1.0 documentation. https://eemeter.readthedocs.io/.

16. EEweather: Weather station wrangling for EEmeter — eeweather 0.3.23 documentation. https://eeweather.readthedocs.io/en/latest/.

17. Kim, Y. J., Lee, S. J., Jin, H. S., Suh, I. A. & Song, S. Y. Comparison of linear and nonlinear statistical models for analyzing determinants of residential energy consumption. *Energy and Buildings* **223**, 110226 (2020).
