# Supplementary material for: Unveiling hidden energy poverty using the energy equity gap
Source: Nat Commun. 2022 May 4;13:2456. doi: 10.1038/s41467-022-30146-5 (PMC9068781; doi:10.1038/s41467-022-30146-5)
Supplement: Supplementary file 3 — Reporting Summary [file 41467_2022_30146_MOESM3_ESM.pdf]

nature portfolio

Corresponding author(s): Destenie Nock, Yueming Qiu

Last updated by author(s): Mar 20, 2022

Reporting Summary

Nature Portfolio wishes to improve the reproducibility of the work that we publish. This form provides structure for consistency and transparency in reporting. For further information on Nature Portfolio policies, see our Editorial Policies and the Editorial Policy Checklist.

Statistics

For all statistical analyses, confirm that the following items are present in the figure legend, table legend, main text, or Methods section.

n/a	Confirmed	
☐	☒	The exact sample size (n) for each experimental group/condition, given as a discrete number and unit of measurement
☒	☐	A statement on whether measurements were taken from distinct samples or whether the same sample was measured repeatedly
☐	☒	The statistical test(s) used AND whether they are one- or two-sided<br>*Only common tests should be described solely by name; describe more complex techniques in the Methods section.*
☒	☐	A description of all covariates tested
☒	☐	A description of any assumptions or corrections, such as tests of normality and adjustment for multiple comparisons
☐	☒	A full description of the statistical parameters including central tendency (e.g. means) or other basic estimates (e.g. regression coefficient) AND variation (e.g. standard deviation) or associated estimates of uncertainty (e.g. confidence intervals)
☐	☒	For null hypothesis testing, the test statistic (e.g. F, t, r) with confidence intervals, effect sizes, degrees of freedom and P value noted<br>*Give P values as exact values whenever suitable.*
☒	☐	For Bayesian analysis, information on the choice of priors and Markov chain Monte Carlo settings
☒	☐	For hierarchical and complex designs, identification of the appropriate level for tests and full reporting of outcomes
☒	☐	Estimates of effect sizes (e.g. Cohen's d, Pearson's r), indicating how they were calculated

Our web collection on statistics for biologists contains articles on many of the points above.

Software and code

Policy information about availability of computer code

Data collection	The household-level electricity consumption and corresponding survey data was provided by the Salt River Project, no code was used in the process. The daily mean outdoor temperature was web scraped using Python 3.8.5, the code can be found here: https://github.com/Pa223/The-Energy-Equity-Gap/blob/main/web%20scraping%20temperature%20data.py
Data analysis	Data processing was done using Python 3.8.5. Data analysis was done using Python 3.8.5 and R 4.0.3. The code and descriptions can be found here: https://github.com/Pa223/The-Energy-Equity-Gap/releases/tag/3%2F18%2F2022

For manuscripts utilizing custom algorithms or software that are central to the research but not yet described in published literature, software must be made available to editors and reviewers. We strongly encourage code deposition in a community repository (e.g. GitHub). See the Nature Portfolio guidelines for submitting code & software for further information.

Data

Policy information about availability of data

All manuscripts must include a data availability statement. This statement should provide the following information, where applicable:

- Accession codes, unique identifiers, or web links for publicly available datasets
- A description of any restrictions on data availability
- For clinical datasets or third party data, please ensure that the statement adheres to our policy

Records of mean daily outdoor temperatures were retrieved from WeatherForYou.com by way of web scraping. The electricity consumption and survey data are from the Salt River Project (SRP). The electricity consumption and survey data are restricted by a non-disclosure agreement. This information is available from the authors upon reasonable request and with permission from the SRP.

Field-specific reporting

Please select the one below that is the best fit for your research. If you are not sure, read the appropriate sections before making your selection.

☐ Life sciences ☒ Behavioural & social sciences ☐ Ecological, evolutionary & environmental sciences

For a reference copy of the document with all sections, see nature.com/documents/nr-reporting-summary-flat.pdf

Behavioural & social sciences study design

All studies must disclose on these points even when the disclosure is negative.

Study description	We use household-level daily electricity consumption data and daily mean outdoor temperature to model the temperature at which each household turns on their air conditioning, the inflection temperature. We then group the households by income and compare median inflection temperatures of each income group to illustrate the inequity in energy poverty and electricity usage. The household-level electricity consumption data is quantitative, the corresponding household surveys are a mixed of quantitative and qualitative data, the outdoor temperature data is quantitative.
Research sample	The sample data was provided by the Salt River Project. The household-level electricity consumption data are of SRP customers living in Phoenix metropolitan, Arizona, USA. The raw data is household-level hourly electricity consumption (kWh/hour), which we consolidated into daily consumption (kWh/day). Each household in the sample data completed a corresponding household characteristic survey, including demographics (race/ethnicity, age), socioeconomic status (income group), and residence characteristics (type of residence, size of residence, age of residence, number of people in household). This is the sample of all consumers that answered the survey conducted by the utility company. The survey provides important technology and socio-demographic information that is essential for our analysis. Our sample is fairly representative of Phoenix, Arizona in terms of race and ethnicity, where the sample distribution of White, Black, Asian, Hispanic, and other ethnicities are 71.2%, 3.5%, 3.9%, 16.7%, and 4.8%, respectively; where data from https://datausa.io/profile/geo/phoenix-az tells us the population is represented as 73.4%, 7.2%, 4.0%, 9.0%, and 6.4%, respectively. The Black population is slightly underrepresented in our sample, and the Hispanic population is over represented, but the sample is large enough that we have enough data to perform statistical testing. The median income of Phoenix reported by census.gov is $60,914 in 2020 dollars, which falls into our most populous bin in our sample ($50,000-$74,999).
Sampling strategy	The utility company randomly selected its residential customers to survey. The survey response rate was about 19%. In the end, there were 13,159 customers who filled out the survey, forming the sample in our paper. This sample is sufficient for our analysis because the sample size is large and also there are enough variations in terms of socio-demographics and technology attributes for us to conduct statistical analyses
Data collection	This is an existing dataset from a utility company, which has metered electricity consumption data collected at the hourly mark across all four years of this study for individual households. The survey was conducted online or by mail. Customers directly filled out the survey online or by hand and mailed them back to the utility company. Weather data was collected by way of webscraping from WeatherForYou.com. Weather data can be found here: https://github.com/Pa223/The-Energy-Equity-Gap/releases/tag/3%2F18%2F2022
Timing	We had four years worth of electricity consumption data, each year is measured from May 1st to April 30th. The dataset starts from May 1, 2015 and ends on April 30, 2019.
Data exclusions	The energy equity gap is measured across income and minority groups, meaning that households that did not report income or race/ethnicity were excluded. For each year of electricity consumption and temperature data, those whose inflection temperature could not be calculated due to incomplete data were excluded, leaving us with 6,002 out of 13,159 usable households in the final analyses.
Non-participation	No participants were involved in this study.
Randomization	Households were grouped by income bracket, then we analyzed the median inflection temperature of each income group to illustrate the difference in electricity consumption behavior between income groups.

Reporting for specific materials, systems and methods

We require information from authors about some types of materials, experimental systems and methods used in many studies. Here, indicate whether each material, system or method listed is relevant to your study. If you are not sure if a list item applies to your research, read the appropriate section before selecting a response.

Materials & experimental systems

n/a	Involved in the study
☒	☐ Antibodies
☒	☐ Eukaryotic cell lines
☒	☐ Palaeontology and archaeology
☒	☐ Animals and other organisms
☒	☐ Human research participants
☒	☐ Clinical data
☒	☐ Dual use research of concern

Methods

n/a	Involved in the study
☒	☐ ChIP-seq
☒	☐ Flow cytometry
☒	☐ MRI-based neuroimaging
